# Radiation Detection—CD/DVD, Glass, and Emerging Materials for Radon Exposure Assessment

**DOI:** 10.3390/s24237674

**Published:** 2024-11-30

**Authors:** Phoka C. Rathebe, Mota Kholopo

**Affiliations:** Department of Environmental Health, Faculty of Health Sciences, Doornfontein Campus, University of Johannesburg, P.O. Box 524, Johannesburg 2006, South Africa; motaliok@gmail.com

**Keywords:** radon detection, CD/DVDs, glass-based detections, alpha track detectors, public health, retrospective radon mapping, lung cancer, nanotechnology

## Abstract

This review aimed to explore advances in radon detection methods, emphasizing cost-effectiveness and accessible techniques such as CDs, DVDs, and glass-based detectors. In this review, we compared traditional methods like alpha track detectors and continuous radon monitors with emerging innovations that leverage polycarbonate material and IoT-integrated systems. Our evaluation of the synthesis suggests that CDs and DVDs provide scalable solutions for long-term radon monitoring, while glass-based detectors like CR-39 offer high sensitivity for epidemiological studies. The integration of IoT and AI technologies further enhances real-time radon monitoring, paving the way for precise, scalable, and affordable radon mitigation strategies. This work highlights the importance of low-cost, innovative approaches in reducing radon-related lung cancer risks and informs future research on optimizing the technologies for diverse environments.

## 1. Introduction

Radon is a naturally occurring radioactive gas that forms from the decay of uranium, thorium, and radium in soil, rock, and water. It enters buildings through cracks and openings, accumulating to harmful levels indoors, particularly in basements and poorly ventilated spaces. Due to its colorless, tasteless, and odorless nature, radon is often unnoticed or undetected without specialized equipment, posing a serious threat to human health. Approximately 40 isotopes of radon are identified, the majority which possess short half-lives ranging from microseconds to milliseconds and hold minimal practical relevance. Radon-222 (radon) and radon-220 (thoron) hold practical relevance. Radon-222, the predominant isotope of radon, with a physical half-life of 3.823 days, results from the decay of radium. Thoron, originating from the decay of thorium, has a physical half-life of 55.6 s [1]. As these isotopes undergo radioactive decay, they release alpha particles, transforming into different elements, known as radon progeny. Radon and its progeny (such as polonium-218 and polonium-214) can enter the human body through inhalation or ingestion. While most of the inhaled radon is exhaled, a small portion, along with its progeny, may remain in the lungs. There, they continue to decay and emit alpha particles [2].

Prolonged inhalation of these radioactive particles can harm the cells lining the lungs [3]. The primary health risk associated with radon exposure is lung cancer. Radon is the second largest cause of lung cancer, behind smoking [4]. Research suggests that radon exposure contributes to 3–14% of all lung cancer cases depending on the average radon levels and smoking prevalence in a given population [5]. Analysis of pooled case–control studies from Europe and North America found that residential radon exposure increases the risk of lung cancer by 8–16% for every 100 Bq/m^3^ increase in radon concentration [6]. Another study in northwestern Spain found that individuals exposed to radon concentration above 50 Bq/m^3^ had a significantly increased risk of lung cancer, with those exposed over 200 Bq/m^3^ having more than double the risk. The synergistic impact of radon exposure and smoking was also evident, as individuals who were heavy smokers and exposed to elevated radon levels faced up to a 29-fold higher risk of developing lung cancer compared to non-smokers with lower radon exposure [7].

With radon being a significant risk factor for lung cancer, especially in confined environments like homes, there is a growing need for innovative, low-cost, and accurate radon detection methods. Radon detection methods are broadly categorized into real-time monitoring methods, providing continuous and immediate data, and periodic monitoring methods, which measure cumulative radon exposure over a defined period. Researchers have recently explored alternative materials such as CDs, DVDs, and glass for radon detection due to their availability, low cost, and high radon absorption properties. The use of CDs, DVDs, and glass materials for radon detection represents a significant advancement in making radon more accessible and cost-effective. These materials and emerging etching techniques provide reliable and accurate results, making them valuable tools in reducing radon-related health risks, particularly lung cancer. Their widespread availability and low cost allow for more comprehensive radon assessment in homes and workplaces, contributing to public health and safety efforts. This review examines advancements in radon detection technologies, focusing on non-traditional materials and their practical applications. It highlights how these innovations address challenges in affordability, accessibility, and scalability, contributing to global efforts to reduce radon-related health risks. By exploring the strengths and limitations of various methods, this article provides a foundation for optimizing radon detection and supporting public health initiatives.

## 2. Methodology

This review evaluates advancements in radon methods, with a focus on accessible and cost-effective solutions such as CDs, DVDs, and glass-based detectors. Scientific articles were retrieved from databases using keywords such as “radon detection”, “CD/DVD radon detectors”, and “glass-based radon monitoring”. The analysis categorized methods into real-time monitoring and periodic monitoring approaches, focusing on their sensitivity, cost, and applicability across diverse environments. Well-established periodic methods like alpha track detectors were compared with innovative techniques like polycarbonate-based CDs and CR-39 glass detectors. Data on each method’s performance in terms of sensitivity, cost-effectiveness, and environmental adaptability were systematically extracted and summarized in comparative tables. Additionally, case studies in residential, industrial, and energy-efficient building settings highlight the practical applications and effectiveness of these methods in various scenarios.

## 3. Periodic vs. Real-Time Radon Monitoring Methods

Radon detection methods can be categorized into two distinct types based on their operational functionality: real-time monitoring methods and periodic monitoring methods.

Real-time monitoring methods provide continuous, instantaneous radon concentration data for immediate use in decision-making and interventions. These methods are particularly useful in dynamic environments where radon levels may fluctuate significantly over short periods. Examples of real-time monitoring methods include continuous radon monitors (CRMs), optical detection systems, and advanced nanotechnology-based sensors, which deliver accurate and timely radon level measurements. These methods are widely regarded for their reliability and efficiency in professional settings such as workplace monitoring and public health surveillance.

Periodic monitoring methods measure radon exposure cumulatively over a defined period, requiring post-exposure analysis. Real-time monitoring methods provide continuous and immediate data on radon levels, enabling timely interventions (e.g., continuous radon monitors and nanotechnology-based sensors).

Periodic monitoring methods, on the other hand, measure cumulative radon exposure over a specified period and require post-exposure analysis. These methods are typically employed for retrospective assessments of long-term monitoring. Established techniques like alpha track detectors and charcoal canisters fall under this category, as do new approaches such as CD-/DVD-based detection and glass-based detectors [8]. These methods are cost-effective and accessible, making them ideal for large-scale public health campaigns or residential monitoring. However, they do not provide feedback, limiting their use in scenarios requiring an immediate response.

While periodic monitoring methods are effective for cumulative exposure assessment, real-time monitoring methods excel in providing actionable data for immediate radon mitigation. For instance, CRMD continuously measures radon levels, offering near-instantaneous results that enable timely interventions [9]. Similarly, optical detection techniques and advanced nanotechnology-based sensors have been developed to enhance sensitivity and tracking capabilities, rivaling or exceeding traditional CRSs in some applications.

### 3.1. Non-Traditional Innovations in Monitoring

Recent advancements in materials and technology have introduced non-traditional methods that challenge the conventional periodic and real-time dichotomy. An example is the CD-/DVD-based detection methods that utilize the polycarbonate layers of disks to capture radon progeny, offering a low-cost solution for cumulative radon assessment. Similarly, glass-based detectors such as CR-39 detectors enable highly sensitive retrospective radon exposure analysis, which is invaluable for long-term epidemiological studies [10]. Some non-traditional methods, like nanotechnology-based sensors, bridge the gap between periodic and real-time monitoring by combining advanced materials (e.g., graphene or carbon nanotubes) with real-time electronic feedback capabilities. These innovations aim to overcome limitations in sensitivity, portability, and affordability, paving the way for more versatile radon detection solutions [11].

### 3.2. Traditional Methods

Traditional radon detection methods, including alpha track detectors, charcoal canisters, and continuous radon monitors (CRMs) have been widely used for decades. These methods are renowned for their simplicity, reliability, and cost-effectiveness. However, these traditional techniques often lack the advanced features of newer methods such as higher sensitivity or adaptability to environmental changes.

### 3.3. Comparative Advantages

While periodic methods like alpha track detectors and CD-/DVD-based detection excel in affordability and cumulative assessment, real-time methods like CRMs and optical detectors provide critical data of immediate action. Each approach serves specific purposes with periodic monitoring offering cost-effective long-term solutions and real-time monitoring addressing radon variations. In summary, the categorization into real-time and periodic monitoring methods highlights the functional distinctions between radon detection approaches, emphasizing their complementary roles in mitigating radon exposure risks. Table 1 below illustrates a comparison of radon detection methods across these categories.

## 4. CD and DVD Optical Methods for Radon Detection

### 4.1. Mechanism of Radon Detection Using CDs and DVDs

**Figure 1:**
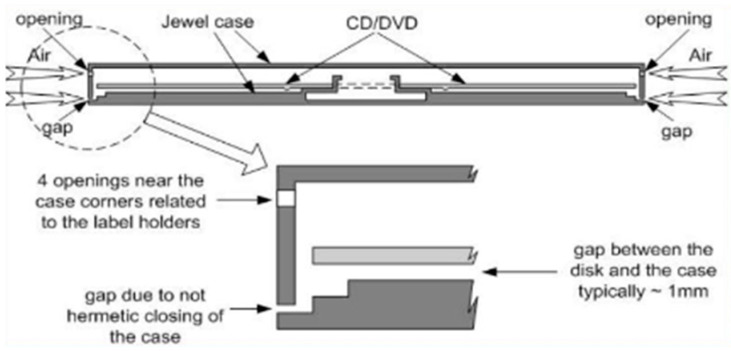
Radon penetration and detection mechanism in CDs/DVDs.

The radon detection process using compact disks (CDs) and digital versatile disks (DVDs) is based on polycarbonate material that forms the core of these disks [8]. Polycarbonate has the ability to absorb gas from the surrounding environment (Figure 1). The jewel case design, with small gaps and openings at the corners and edges, facilitates the entry of radon gas into the case. Once inside, the gas interacts with the polycarbonate layer of the disk.

When radon-222 decays, it emits high-energy alpha particles, which interact with the polycarbonate, leaving latent permanent damage tracks that can only be reversed through specific etching techniques (electrochemical etching). The polycarbonate material is ideal as it retains these alpha tracks over long periods even without continuous exposure to radon. Significant research including that conducted by Pressyanov et. al. [8] demonstrates that alpha particles emitted by radon generate discernable imprints or track beneath the surfaces of CDs and DVDs. This facilitates a retrospective assessment of radon exposure, as the quantity of tracks is directly proportional to the environmental concentration of radon over time. The alpha tracks are exposed through etching at designated depths generally between 69 and 80 µm, allowing for the measurement of radon concentrations without interference from other environmental variables [18]. This depth enables differentiation between radon-222 (the most common isotope for radon-related health hazards) and thoron (thoron 220). Thoron decays faster than radon 222 which implies its alpha emissions are detected at a shallower depth.

The electrochemical etching technique entails submerging the disk in an electrolytic solution and applying a regulated electrical current, usually direct current (DC), which creates an oxidation reaction on the disk surface. This enlarges the dimensions of the alpha-induced tracks, rendering them observable under a microscope or with specialized optical apparatus. The quantity of tracks correlates with the duration of radon exposure, rendering CDs and DVDs appropriate for retrospective assessment [19].

### 4.2. Comparative Efficiency of CDs and DVDs as Radon Detectors

The efficiency of CDs and DVDs for radon detection depends on several factors, including material thickness, etching conditions, and the polycarbonate material’s radon absorption capacity. DVDs are noted for their ability to handle higher radon concentrations and environmental conditions, making them more suitable for applications in high-risk or industrial settings. Table 2 below provides the comparative efficiency of CDs and DVDs as radon detectors.

The CD/DVD radon detection method presents a unique and valuable approach for retrospective and long-term radon measurements as it is a passive method of cumulative exposure rather than real-time monitoring. The superior performance of DVDs compared to CDs in high radon concentrations and varied environmental conditions can be attributed to their multilayer polycarbonate structure. Unlike CDs, which use single-layer polycarbonate, DVDs feature multiple polycarbonate layers that provide greater capacity to absorb and retain alpha particle tracks. This reduces the likelihood of track saturation, a common issue in high-radon environments where concentrations may exceed 400 Bq/m^3^, making them particularly suitable for industrial applications or areas with significant radon emissions.

Further, the structural integrity of DVDs enhances their adaptability to environmental fluctuation. Variations in temperature and humidity can significantly affect the accuracy of radon detection. DVD multilayer design minimizes the impact of these external conditions by providing greater stability and reducing measurement variability. This makes DVDs a more reliable option for environments with unstable climate conditions, such as underground mines or industrial facilities with varying ventilation. These advantages, combined with their widespread availability and cost-effectiveness, highlight DVDs’ potential as a robust tool for radon detection in diverse settings. While CDs remain the practical choice for general radon mapping, DVDs’ enhanced properties make them the preferred option for high-radon environments and critical industrial applications.

While DVDs demonstrate superior performance in high-radon and variable environmental conditions, CDs offer distinct benefits for general radon detection. Their single-layer polycarbonate structure simplifies post-processing and reduces calibration requirements, making them more accessible for non-specialist users. CDs are particularly effective for detecting radon levels within a typical residential range of 20–200 Bq/m^3^, covering the majority of environments where extreme sensitivity is unnecessary. Furthermore, their slightly lower cost, while minor for individual detectors, becomes a significant advantage in large-scale projects, such as public health campaigns or community radon mapping efforts. These attributes make CDs a practical and economical choice for widespread deployment in standard radon detection scenarios.

### 4.3. Application and Case Studies Using CD/DVD Methods

Radon Mapping: CDs and DVDs have been effectively used for large-scale radon mapping projects. A significant case study is the radon mapping project conducted in the suburbs of Sofia, Bulgaria, where CDs and DVDs from 462 homes were analyzed for radon exposure. The results revealed that radon concentrations in homes varied significantly with some districts showing 74% of homes exceeding recommended levels of 300 Bq/m^3^ [25]. This method allowed for the identification of radon priority areas where further mitigation measures were needed.

Energy-Efficient Buildings: CDs and DVDs are used to monitor changes in radon levels after energy-efficient building renovations as modern building measures aimed at enhancing energy efficiency can unintentionally raise indoor radon concentrations by trapping radon gas inside. In a study by Pressyanov et al. [8], CDs and DVDs were used to monitor radon levels in homes that had undergone energy-efficient renovations. The results showed that 35% of the renovated homes experienced a significant increase in radon levels [19]. The case highlights the importance of using retrospective detection, especially in homes that have not been previously monitored but are now at greater risk due to changes in design.

Mining and Industrial Applications: CDs and DVDs have also been used in industrial settings, particularly with high radon concentrations, such as underground mines. In a Bulgarian mine study, CDs and DVDs were employed as passive detectors to monitor radon and thoron levels in mining shafts [20]. The disks provided accurate radon measurements and assisted in the identification of ventilation issues by detecting areas with inadequate airflow where radon accumulated.

## 5. Glass-Based Radon Detection

### 5.1. Mechanisms and Effectiveness

The glass-based radon detection method involves the use of standard or specialized glass, which interacts with ionizing radiation emitted by radon decay products. This interaction leaves tracks on the surface, which can be analyzed through chemical etching. This method leverages the glass’s ability to retain these tracks, facilitating retrospective radon exposure assessments without requiring continuous power [10,26]. In contrast, the CR-39 detection technique, categorized as a solid-state nuclear track detector (SSNTD), employs a polymer material uniquely sensitive to alpha particles. CR-39′s structure allows for the recording of radon progeny with greater precision, making it ideal for studies needing highly sensitive and specific measurements [10]. The CR-39 method also utilizes a specialized etching process that exposes alpha particle tracks in a way that is more refined and countable than glass, enhancing measurement accuracy [15]. While both methods rely on track etching to visualize radon exposure, the CR-39 detector’s sensitivity and chemical durability make it preferable for epidemiological applications, whereas glass-based methods are often used in broader, long-term retrospective assessments [26].

### 5.2. Performance and Application of Glass-Based Radon Detectors

Glass-based radon detectors, particularly those using CR-39, are highly effective tools for radon monitoring due to their unique combination of sensitivity, cost-effectiveness, and versatility. These detectors excel in measuring cumulative radon exposure assessments. Their sensitivity allows them to detect low radon concentrations, making them suitable for residential, workplace, and environmental studies. A key advantage of glass-based detectors is their ability to store historical radon exposure data on glass surfaces without requiring active power sources. This feature is particularly beneficial in epidemiological studies where long-term exposure assessments are necessary to link radon exposure with health outcomes, such as lung cancer. Additionally, the cost-effectiveness of CR-39 detectors makes them a practical option for large-scale monitoring programs, especially in resource-limited settings.

Glass-based radon detectors are highly sensitive and effective for long-term radon monitoring, but they have limitations, such as reduced counting efficiency in high-radon areas due to overlapping alpha tracks and sensitivity to environmental factors like temperature and pressure. The need for post-processing, such as chemical etching, adds complexity compared to real-time systems [15,27]. These detectors have diverse applications including geological studies for radon emissions along fault lines, seasonal assessments in homes, and uranium exploration through radon detection in soil and rocks [10,28]. Despite these challenges, their cost-effectiveness and versatility make them vital for radon detection in various environments [15,29].

### 5.3. Application and Case Studies

Retrospective Radon Exposure Assessment in Homes: The most impactful application of CR-39 detectors is the ability to retrospectively assess retrospective residential exposure environments. Radon progeny, particularly polonium isotopes (210Po), settle into glass surfaces over time due to alpha decay. This facilitates the assessment of historical radon levels in residences, regardless of current radon concentrations. The capacity to assess radon exposure longitudinally renders these detectors highly valuable for epidemiological studies seeking to link prolonged radon exposure with health outcomes such as lung cancer. A study in Germany’s Schneeberg–Schlema region utilized the CR-LR difference techniques, a method based on the CR-39 and LR-115 detectors. This method evaluated historical radon concentrations in residences situated in homes located in former uranium mining districts. Radon levels from past decades were estimated to be up to 50 times higher than current levels, offering essential insights into the long-term health concerns encountered by residents in these regions [30].

Seismic Activity Monitoring and Earthquake Prediction: Glass-based radon detectors, particularly CR-39 solid-state nuclear track detectors, are increasingly used in geographical research to monitor radon emissions in seismic zones. The identification of increased radon levels in the soil is associated with tectonic activity, positioning radon detection as a proactive instrument for earthquake forecasting. Radon gas can permeate fault lines and fissures in the Earth’s crust, and variations in radon concentrations frequently precede seismic occurrences. In a study conducted on the Pernicana fault system at Mount Etna, Italy, researchers used CR-39 detectors to measure radon emissions along tectonic faults. The study revealed that radon concentrations were higher in specific locations, correlating with known geological and tectonic activity. These findings suggest the reliability of glass-based detectors as a tool for monitoring soil radiation with active faults [28].

Indoor Radon Monitoring for Public Health: CR-39 detectors are used widely in indoor radon monitoring to assess radon concentrations in schools, homes, and workplaces. Long-term exposure is a significant public health concern as radon is the second leading cause of lung cancer. The effectiveness of CR-39 detectors in providing reliable data on seasonal and spatial variations in radon concentrations was proven in a Swaziland study by Mahlobo et al. [31] where CR-39 detectors were used to measure indoor radon levels in 57 homes across different building types. The results indicated an average concentration of 69 Bq/m^3^ with higher levels in winter due to reduced ventilation. Ground-floor homes had significantly higher radon concentrations compared to upper floors. Radon Detection in Uranium and Exploration: Radon is an important indicator of uranium deposit as it is a decay product of uranium. CR-39 detectors are often used in uranium exploration to monitor radon levels in soil and rocks, providing geologists with information on potential mining sites.

In retrospective research conducted in former uranium mining locations, CR-39 detectors were utilized to evaluate historical radon levels from glass surfaces in residences placed near mining sites. This study indicated that dwellings in mining districts had much higher historical radon concentrations owing to uranium decay, providing evidence of the long-term exposure dangers for inhabitants living near these sites [10]. These findings underscore the necessity of radon detection in both public health and geological research situations. Retrospective Assessment of Radon in Epidemiological Studies: In an epidemiological study, retrospective radon exposure assessment using CR-39 detectors fitted to glass surfaces allows researchers to estimate radon levels in past decades. This method is important in investigating the long-term health impacts of radon exposure, particularly where radon monitoring was not historically undertaken. A study in the United States was conducted in which CR-39 detectors were attached to household glass objects to measure residential decay products, enabling estimation of the past cumulative radon exposure in homes. The results were used to fill gaps in exposure for subjects in a lung cancer epidemiological study, and the method was found to correlate well with year-long ambient radon measurements [29]. The study demonstrated the effectiveness of CR-39 detectors in enhancing the accuracy of exposure data in radon epidemiology.

## 6. Incorporating Novel Materials: Nanotechnology and Polymers in Radon Detection

The exploration of nanomaterials and advanced polymers in radon detection presents new avenues for enhancing sensitivity and cost-effectiveness. These materials, particularly graphene-based nanocomposites and carbon nanotubes (CNTs), offer properties such as high surface area and improved electronic response, which are valuable for designing highly sensitive sensors. Although radon detection using nanotubes is an emerging field, preliminary studies show potential in leveraging these materials for low-level detection, with ongoing research seeking to validate their efficacy specifically in radon detection scenarios [11,32].

### 6.1. Polymers in Radon Detection

Functional polymers, such as polydopamine-coated surfaces, are being developed for selective detection of radon decay products. Unlike traditional methods relying on physical adsorption, a polymer can be chemically engineered to bind radon progeny specifically, enhancing detection precision [33]. This selectivity and rapid response potential make polymer sensors promising for diverse radon monitoring applications, especially in environments where traditional methods may be limited by environmental interference.

### 6.2. Comparative Analysis of Novel Material vs. Traditional Techniques

Compared to traditional materials like CD/DVD polycarbonate layers and CR-39 glass detectors, nanomaterials and polymers offer high sensitivity, faster response time, and improved portability. Table 3 below summarizes these properties, illustrating the potential of nanotechnology and polymer-based sensors to overcome limitations seen in passive radon measurement methods.

## 7. Economic Feasibility and Practical Applications of CDs, DVDs, and Glass-Based Radon Detection

The accessibility and low-cost materials for CDs, DVDs, and glass-based detectors make them viable for widespread radon monitoring in homes, schools, and workplaces. These materials support DIY radon detection kits and large-scale radon mapping projects, especially in regions with limited resources. Studies in Spain have shown the utility of such systems in effectively monitoring indoor radon levels at low cost, making radon detection more accessible to the public [36].

### Affordability and Accessibility

CD- and DVD-based radon detectors are particularly beneficial for community health programs due to their affordability and ease of use. They provide a cost-effective detector, allowing for extensive public health monitoring and mitigation in high-risk areas. Glass-based systems, particularly those using CR-39, are also economically feasible and have been effectively utilized in home-based radon monitoring [36].

## 8. Technological Integration: IoT and AI in Radon Monitoring

The integration of Internet of Things (IoT) technologies and artificial intelligence (AI) has revolutionized radon monitoring by enabling real-time data acquisition, analysis, and intervention. IoT-enabled radon sensors can continuously track radon levels in various environments, triggering alerts when radon exceeds safe limits, thereby enabling timely interventions. Smart home radon detectors, combined with IoT systems, contribute to improved indoor air quality by supporting real-time detection and automatic mitigation systems such as ventilation controls [37].

### Machine Learning for Enhanced Detection Accuracy

Artificial intelligence (AI) and machine learning (ML) models, such as Random Forest algorithms, are increasingly being used to analyze radon data from IoT sensors, allowing for accurate trend detection and early warning. These advancements support targeted public health interventions by identifying high-risk radon exposure areas and predicting seasonal variations, which can help mitigate lung cancer risk associated with long-term radon exposure.

## 9. Environmental and Ecological Impact of Non-Traditional Radon Detection Methods

The shift toward using eco-friendly and recycled materials, like glass and repurposed CDs/DVDs, offers environmental benefits by reducing waste and resource consumption. These materials are also compatible with sustainable building practices, supporting green certifications such as LEED by contributing to better indoor quality without requiring resource-intensive materials [38].

### Green Building Applications

Eco-friendly radon detection technologies can be integrated into green buildings, supporting sustainability by improving indoor air quality monitoring. The use of recycled glass or CDs aligns with sustainable architecture principles, helping minimize environmental impact on radon detection.

## 10. Health Risk Modeling and Predictive Analysis Using Non-Traditional Methods

Advanced radon detection materials, such as CR-39 glass detectors, allow for precise health risk assessment by providing long-term radon exposure data. These detectors contribute to epidemiological studies by offering cumulative exposure information which is crucial in estimating lung cancer risk related to radon. Combining deep learning models with radon exposure data enables accurate risk stratification, improving early detection and intervention for radon-related health threats [26].

## 11. Conclusions

This review highlights the advancements in non-traditional radon detection methods, emphasizing the cost-effectiveness, sensitivity, and public health benefits of materials such as CDs, DVDs, and CR-39 glass detectors. These materials allow for more accessible radon monitoring, supporting large-scale health initiatives and enabling detection in diverse environments. By integrating IoT and AI technologies, these non-traditional methods further improve detection accuracy and real-time response capabilities, ultimately contributing to reduced radon exposure and lung cancer risk. Future research should focus on optimizing these materials for varied environmental conditions and on further integrating smart technologies to advance radon detection and mitigation.

## Figures and Tables

**Table 1:** Comparison of real-time and periodic radon monitoring methods.

Detection Method	Category	Advantages	Limitations	References
Alpha Track Detectors	Periodic Monitoring	Relatively affordable, straightforward chemical processing for track reading	Limited to long-term, post-exposure analysis only; requires lab setup for processing	[12]
Charcoal Canisters	Periodic Monitoring	Short-term, simple, cost-effective	Susceptible to environmental factors; requires analysis after exposure period	[13]
Continuous Radon Monitors (CRMs)	Real-Time Monitoring	Real-time data acquisition, accurate, commonly used in professional settings	Higher costs; requires professional handling and calibration	[9]
Electret Ion Chambers	Periodic Monitoring	Short- and long-term, cost-effective	Requires special equipment for analysis	[13]
CD-/DVD-Based Detection	Periodic Monitoring	Extremely low cost due to the widespread CD/DVD availability, allows for DIY and scalable monitoring	Requires more complex post-exposure electrochemical etching; not widely adopted yet	[14]
Glass-Based Detection	Periodic Monitoring	Highly sensitive, for long-term monitoring, suitable for historical exposure assessment	Affected by environmental factors (temperature, pressure); requires specialized processing for track reading	[15]
Aptamer-Based Detection	Real-Time Monitoring	Highly sensitive, non-radioactive	Complexity in design and application	[16]
Optical Detection Methods	Real-Time Monitoring	Rapid, real-time detection	Requires optical systems and calibration	[14]
Two-Filter Dual-Flow-Loop Monitors	Real-Time Monitoring	Continuous, highly accurate, portable	Complex and costly, requires specialized calibration	[17]

**Table 2:** Comparative analysis of CDs and DVDs as radon detectors based on efficiency and application.

Criteria	CDs	DVDs	Reference
Material Thickness	Single-layer polycarbonate	Multilayer polycarbonate	[20]
Alpha Track Depth Detection	69–80 µm (shallow depth)	Up to 120 µm (deeper detection)	[18]
Detection Sensitivity (Radon Concentration Range)	Reliable for 20–200 Bq/m^3^	Effective for 20–600 Bq/m^3^	[19]
Minimum Detection Limit	~20 Bq/m^3^	~15 Bq/m^3^	[21]
Etching Process Sensitivity	Moderate, optimized etching needed	High sensitivity due to multilayer structure	[22]
Performance in High Concentrations	May saturate at >400 Bq/m^3^	Reliable in concentrations > 500 Bq/m^3^	[23]
Environmental Condition Sensitivity	Minor variability in efficiency under stable conditions	Better adaptability to temperature/humidity changes	[24]
Cost	Low (widely available, affordable)	Low (slightly higher than CDs)	[21]
Application	Suitable for general radon mapping, mining, homes, and workplaces	Optimized for high-radon environments such as industrial settings	[20]
Long-Term Retrospective Measurement Capacity	Effective for extended measurements (months), reliable for long-term retrospective measurement	Suitable for both short-term and long-term measurements	[25]

**Table 3:** Comparative analysis of novel material vs. traditional techniques.

Feature	Nanomaterial (Graphene, CNTs)	Polymers (Functionalized Sensors)	Non-Traditional Techniques (CD, DVD, Glass)
Sensitivity	High sensitivity due to large surface area and electron mobility [11]	Moderate to high sensitivity; selective binding to radon decay particles [33]	Lower sensitivity; relies on physical adsorption or etching by radon progeny
Response Time	Real-time detection with rapid electronic transfer [32]	Near real-time, fast response based on selective chemical interactions	Delayed detection; post-exposure analysis required
Portability	Portable devices with continuous monitoring capabilities [34]	Portable and adaptable to different environmental conditions [33]	Limited portability; typically requires fixed systems
Selectivity	High selectivity with functionalized surfaces and nanocomposites [35]	Selective binding of radon decay products through chemical engineering	Lower selectivity; relies on physical interaction with radon progeny
Environmental Adaptability	Effective in various environments; durable materials with minimal interference	Highly adaptable to diverse environmental conditions (temperature and humidity)	Affected by environmental factors like humidity and temperature
Cost	Moderate to high due to advanced materials and fabrication costs	Moderate; potentially cost-effective in mass production	Generally low-cost methods with limited precision and functionality
Maintenance and Calibration	Requires periodic calibration; high-precision electronics	Requires maintenance for surface coating and functional materials	Requires maintenance for surface coating and functional materials
Scalability	High scalability for industrial applications and large-scale monitoring	Highly scalable, especially in environmental monitoring systems	Limited scalability due to size and post-processing requirements

## Data Availability

Not applicable.
